# Evaluation of the Effectiveness of Active and Passive Safety Measures in Preventing Ship–Bridge Collision

**DOI:** 10.3390/s22082857

**Published:** 2022-04-08

**Authors:** Wenqing Ma, Yini Zhu, Manel Grifoll, Guiyun Liu, Pengjun Zheng

**Affiliations:** 1 Faculty of Maritime and Transportation, Ningbo University, Ningbo 315832, China; 1911084005@nbu.edu.cn (W.M.); 1911084009@nbu.edu.cn (Y.Z.); 2 Collaborative Innovation Center of Modern Urban Traffic Technologies, Southeast University, Nanjing 211189, China; 3 National Traffic Management Engineering & Technology Research Centre, Ningbo University Sub-Centre, Ningbo 315832, China; 4 Barcelona Innovative Transportation, Barcelona School of Nautical Studies, Universitat Politècnica de Catalunya (UPC—BarcelonaTech), 08003 Barcelona, Spain; manel.grifoll@upc.edu

**Keywords:** ship–bridge collision prevention, safety measure, Monte Carlo simulation, Bayesian networks, AIS data

## Abstract

The risk of ship–bridge collisions should be evaluated using advanced models to consider different anti-collision and bridge-protection measures. This study aimed to propose a method to evaluate the effectiveness of active and passive safety measures in preventing ship–bridge collision. A novel ship–bridge collision probability formulation taking into consideration different safety measures was proposed. The model was applied at Jintang Bridge in China where the surrounding vessel traffic is ultra-crowded. We calculated the collision probability between the bridge and passing traffic using automatic identification system (AIS) data, Monte Carlo simulation, and Bayesian networks. Results under four different safety measures (i.e., active measures, passive measures, both measures and none) were analyzed and compared. The analysis concluded that both active and passive safety measures are effective in reducing the ship–bridge collision probability. Active measures, if deployed properly, can provide protection at an equivalent level than passive measures against collision risks. However, passive measures, such as setting arresting cables, are necessary in cases where the response time of the active measures is long. The proposed method and the results obtained from the case study may be useful for robust and systematic effectiveness evaluation of safety measures in other cases worldwide.

## 1. Introduction

Bridges are at risk of ship contacts or collision, which cause serious damage or even the collapse of bridges. Strictly IMO recommendations refer to collision when there is contact between two moving objects and contact when a ship strikes a bridge pier. However, many academics used collision for pier–ship contact, so ship–bridge collision in this paper refers to the potential contact or strike of two objects between a ship and bridge. According to AASHTO [1], from 1960 to 2002, ship collisions caused the collapse of 31 major bridges worldwide, resulting in 342 deaths. In 1980, the Sunshine Skyway Bridge collided with a ship, killing 35 people. Moreover, nearly 100 collision accidents between ships and bridges were recorded from 1959 to 2008 from the Yangtze River Bridge in Wuhan, China [2].

In 2008, the deck of the under-constructed Jintang Bridge in Ningbo, Zhejiang, China, was struck by the “Qinfeng 128” ship, resulting in one span of the box girder dropping into the water. In this accident, four sailors were killed. The reasons concluded by the accident investigation team are as follows: (1) The crew used uncorrected charts and planned wrong routes and (2) the crew did not fully grasp the navigable conditions of the Jintang Bridge, illegally crossing the non-navigable spans of the bridge, which is the direct cause of the accident.

In 2016, the high-speed passenger ship “UNIVERSAL MK 2017” carried 67 passengers and touched the anti-collision pile of the Hong Kong–Zhuhai–Macao Bridge in the Lingding Waterway of Guangzhou, three anti-collision piles on the north side were deformed. The accident investigation team states the reasons as [3]: (1) The crew did not keep a regular lookout and did not find the ship off course in time and (2) the ship sailed with poor visibility, not using safe speeds, and not navigating cautiously.

In recent years, both ship traffic and hydraulic facilities, such as cross-sea bridges in coastal areas are increasing rapidly [4,5]. This will increase the risk of ship–bridge collision. Anti-collision safety measures are widely used to protect bridges from ship collision. However, previous traditional research and methods only focused on the analysis of anti-collision measures itself, which depends on its strong structure to withstand ship collision to minimize the loss [6].

With the development of anti-collision safety measures, more and more safety measures are proposed to protect bridges including active measures and passive measures.

Passive measures, also known as physical measures, refer to building anti-collision piers and/or adding arresting cables or anti-collision boxes to protect bridges. They can be divided into two types [7]. The first type is setting anti-collision devices to endure the ship collision impact instead of the bridge directly. The anti-collision device is located outside the bridge pier, similar to artificial islands, anti-collision pier, and arresting cables. When the ship accidentally deviates from the fairway and is about to hit the bridge, it will first impact the anti-collision device and absorb part of the impact force. The second type is anti-collision structures directly attached to the bridge pier. When the ship hits the bridge, it will unload the impact force. The common structures are collision steel casing, rubber guard, and cushioning energy materials. However, passive measures cannot prevent collisions and have poor anti-collision effect for large ships. Moreover, passive measures are expensive to build, and they are prone to structural aging and have an impact on the marine environment.

Active safety measures include early warning system and rescue tugboat measures. The early warning system reminds ships of the danger of collision with bridges by relevant technical means before an accident happens, and tugboat rescue can take the ship out of the collision path. Active safety measures are very flexible. Early warning and tugboats may be set up in the first few years of operation of a new bridge to prevent ships from being unfamiliar with the waterway environment, which can be withdraw in all or part after the traffic flow in the bridge area stabilizes when the risk of collision reduces to a certain level.

It is important to evaluate the effectiveness of these different protection measures to determine whether those measures are sufficient and which measure is better. However, the existing studies on passive measures mainly focused on reducing the impact strength to avoid bridge collapse. For example, Moan et al. [8] designed a high strength bracket to penetrate the bow in case of a collision accident. The ship consumed a lot of collision energy through the deformation and damage of the bow, reducing the risk of bridge collapse. Studies on the effectiveness of active safety measures are rare to our knowledge, and there is no comprehensive method available to evaluate and compare the effectiveness of active measures with others.

**Figure 1:**
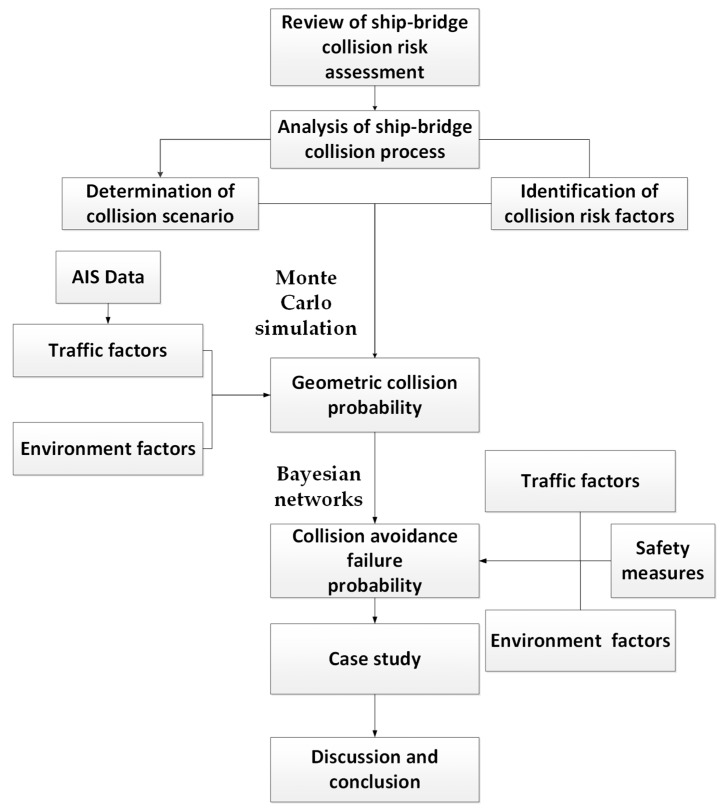
Structure of the paper.

Therefore, this paper proposes a novel method for evaluating the effectiveness of safety measures to avoid ship–bridge collisions. The effectiveness of both active (early warning and setting tugboats for rescue) and passive (setting arresting cables) safety measures are evaluated using the Monte Carlo simulation and Bayesian networks method. The rest of the paper is structured as follows (Figure 1): Following the introduction, Section 2 carries out a comprehensive literature review on ship–bridge collision risk assessment. Section 3 describes the proposed model for calculating the ship–bridge collision probability. In Section 4, the model is used to evaluate the effectiveness of both active and passive measures, and the results are compared. Finally, in Section 5, conclusions and the directions of further research are highlighted.

## 2. Literature Review

### 2.1. Ship–Ship Collision Risk Assessment Research

To review the research related to ship–bridge collision, it is necessary to start from the ship–ship collision research to make a comprehensive grasp of the ship collision risk assessment.

According to different research perspectives, the studies on ship–ship collision risk can be generally divided into micro- and macro-collision risk research.

Micro ship–ship collision risk assessment is a qualitative or quantitative assessment of the collision risk of a single ship or multiple ships in encounter situations based on real-time behavior characteristics of ships. It is a concept in the theory and practice of ship collision avoidance. In the practice of collision avoidance, the quantitative index of collision risk degree is usually called Collision Risk Index (CRI), which is usually between 0 and 1. The risk of collision can provide basis for the crew to take measures to avoid collision.

The most representative studies of micro ship–ship collision risk assessment are based on the ship domain theory [9,10] and studies based on distance to closest point of approach (DCPA) and time to closest point of approach (TCPA). The ship domain was first proposed by Fujii and Tanaka [11], who defined the ship domain as the effective domain around a ship into which other ships avoid entering. DCPA and TCPA are widely used in microscopic collision risk because of their simplicity of interpretation. The DCPA can directly reflect the minimum distance of the two ships at the most dangerous moment. The TCPA directly reflects the urgency of both ships.

Macro ship–ship collision risk assessment refers to the study of the ship–ship collision risk of the whole area of the navigation environment, which is often based on the historical ship–ship collision accident data, traffic flow statistics, related hydro-meteorological data, and expert questionnaire forms. It is within a certain range of navigation safety condition of comprehensive evaluation. Compared with the micro ship–ship collision risk assessment research, the factors considered in the macro ship–ship collision risk assessment are more comprehensive, and most of its methods belong to the idea of probabilistic statistical analysis, which can be summarized as follows:

1. Ship–ship collision risk assessment based on historical collision number or collision rate.

It is the simplest and most primitive way to characterize the risk using the number of ship–ship collisions in a unit of time. The higher the number of ship–ship collisions in a unit of time, the greater the risk of collision in the water area. The ship–ship collision rate is an improvement of the above method, which refers to the ratio of the number of ships involved in a collision in a unit of time and the number of ships passing in a unit of time in the water. However, in theory, ship–ship collision accidents are rare events, and the model of the ship–ship collision number or collision rate based on historical statistics may not reflect the potential ship collision risk when historical data are rare when the environment changes over the time.

2. Ship–ship collision risk assessment based on encounter number or encounter rate.

In the International Regulations for Preventing Collisions at Sea (COLREG), Rule 8 stipulates: “Action taken to avoid collision with another vessel shall be such as to result in passing at a safe distance”. The encounter between ships is the prerequisite for ship–ship collision. Therefore, it is necessary to maintain a certain safe distance during the encounter.

Many scholars have the ship–ship collision risk assessment method based on the encounter number or encounter rate, which can well reflect the potential collision risk, and the encounter is closely related to the actual navigation environment. Therefore, this method is more reasonable and applicable to describe the ship–ship collision risk.

3. Ship–ship collision risk assessment based on collision probability.

Typically, marine accident probabilities are modeled based on the work of Fujii et al. [12] and Macduff [13] in the 1970s. They first proposed the collision probability model and at that time, ship movements were often estimated based on records of radar images. MacDuff [13] in 1974 proposed a method to model the probability of collision accidents during ship navigation. In this model, he proposed that the ship–ship collision probability in the channel can be divided into two parts: geometric probability and causation probability. This also lays the foundation for the study of the ship–bridge collision probability. The geometric collision probability is the probability of collision in the scene where all ships in a certain area use auto pilots (i.e., assuming no one is in control of the rudder) and sail at a certain course and speed. The causation probability indicates the probability of failing to avoid the collision for the ships being on a collision course, mostly due to the error of the navigator or technical fault. The probability of collisions is very sensitive to the causation probability value and it should be modelled with great care to obtain reliable results [14,15,16]. Ship–ship collision risk assessment based on collision probability provides concise and quantitative results for risk assessment, generally only need to input the macroscopic traffic flow characteristics of the ship to obtain the geometric probability, so it is widely used in research. Various scholars have proposed many new models and methods focusing on the ship–ship collision probability model, including the Pedersen [17] model, Fowler and Sorgdrd model [18], and the IWRAP model [19]. However, the collision probability models do not make a big difference with the MacDuff model. The common thinking is to separate the collision probability into two parts: the probability of being on a collision course and the probability of failing to avoid the accident while being on a collision course.

### 2.2. Ship–Bridge Collision Risk Assessment Research

The ship–bridge collision scene can be regarded as a special case of the ship collision scene, that is, the encounter object of the ship is a fixed structure—a pier and in this sense, all the theories of collision avoidance such as the ship domain and DCPA/TCPA can be applied into ship–bridge collision prevention. In the study of ship–bridge collision risk, it is similar to the ship collision risk, which can be divided into micro and macro ship-bridge collision risks according to different research angles.

Micro ship–bridge collision risk assessment is a qualitative or quantitative evaluation of the ship–bridge collision risk during the period where the ship passes the bridge according to the real-time behavior characteristics of the ship and the environment, which is from the perspective of the specific ship.

From the microscopic aspect, Liu et al. [6] proposed a ship–bridge collision hazard model based on collision avoidance theory. Both the DCPA and TCPA are integrated into an index called the degree of collision risk. Wu et al. [20] put forward a ship–bridge collision early warning system based on fuzzy logic. The study focused on collision detection methods and integrated bridge systems for the collision risk assessment considering the uncertainty of the ship state during complex ship exercises. In this study, the authors consider the ship position, ship trajectory direction, ship to bridge distance, and ship speed as the system input and the ship–bridge collision risk is presented after defuzzifying the output. Most of the current studies focus on the macro ship–bridge collision risk to assess the overall risk level of ship collision to the bridge.

At present, there are three main directions in the field of macro ship–bridge collision risk assessment: calculation of ship collision probability, bridge collapse probability, and assessment of ship–bridge collision consequences (risk acceptance criteria). The conclusions of these work can provide guidance for the design of the bridge [21] and a basis for the layout of active and passive anti-collision measures of the operating bridge.

The active collision prevention research of ship–bridge is always developed [22] around non-real-time statistics-based collision probability. At present, bridge risk management mainly focuses on the calculation of ship collision probability, and most of the research was conducted on some specific bridges [23], rather than from the systematic direction. There is no unified understanding of the study of consequence assessment.

Some studies investigated the ship–bridge collision probability through exploratory analysis. Xia [24] analyzed the navigational risks, including the safety distance risk, the pier collision risk, and the traffic congestion risk, in the Nanjing Yangtze River Bridge (NYRB) waters based on spatiotemporal mining on massive AIS trajectories. Pier collision risks were studied by analyzing the spatial relationship between vessels and the bridge. However, the risk is only given by illustrations without quantitative analysis, which limited its applications and cannot be seen as a risk assessment method.

Similar to the ship collision probability study mentioned above, the ship–bridge collision probability calculation methods can be divided into two categories: one is based on the historical collision number and collision rate, and the other is based on collision probability.

However, it is usually difficult to determine the probability based on the historical statistics especially for those newly built bridges since the ship–bridge collision accidents are rare events.

The most widely used method of collision risk assessment is based on the collision probability method. The collision probability method is the modeling method which calculates the ship–bridge collision probability using mathematical formulations, such as the American Association of State Highway and Transportation Officials (AASHTO) [15] model, Eurocode [25] model, Larsen model [26], or KUNZI model [27]. For instance, load and resistance factor design (LRFD), from AASHTO, employs the “annual frequency of bridge collapse” (AF) concept to estimate the risk of bridge collapse, which is the product of the probability of ship–bridge collision and the bridge collapse probability after the collision. Similarly, Eurocode estimates the “probability of overall collapse of bridge” mainly based on the judgment of the engineer [28]. In this regard, the determination of the ship–bridge collision probability is the most important part in the risk assessment [29]. In [30], the authors combined the advantages of several ship–bridge collision models and improved the AASHTO model by using the ship simulator to obtain the track probability distribution curve of the ship to replace the original geometric probability distribution curve. Moreover, it introduced ship stopping probability function to address the shortcomings of the AASHTO model.

Various studies investigated modelling approaches or frameworks for ship–bridge collision risk and usually validated the proposed model by applying the model into a specific area. Axel Horteborn et al. [31] presented a methodology using AIS data, a ship maneuvering simulator, and the Monte Carlo method to calculate the accident probabilities in marine traffic near bridges spanning over wide waterways. Hansen et al. [32] evaluated the ship–bridge collision risk in the Sognefjorden Strait by transforming the available information, including bathymetry, geography, bridge geometry, and AIS data, into a risk model that can estimate ship collision probabilities. Zhang et al. [29] proposed a fully probabilistic framework for assessing the ship–bridge collision risk, and a simplified empirical model for evaluating ship–bridge collision force is then adopted. The probabilistic distribution of the collision force can be obtained through Monte Carlo simulation. Furthermore, the finite element method simulation is conducted to estimate the collapse probability of piers. Park et al. [33] presented a probabilistic design framework for evaluating very rare human-made hazards, such as collisions and explosions. Pedersen et al. [21] proposed a rational design procedure for bridge piers and pylons against ship collision impacts, where the most important part is a procedure for calculating the probability of critical ship encounter situations near the bridge, and the probability of ship collision accidents caused by human errors and technical errors are considered. Yu et al. [34] proposed a novel Bayesian-based model to assess the collision risk between ships and offshore installations (SOI) involving passing ships.

The AASHTO model may be the most widely used model for calculating the ship–bridge collision probability. Here, the annual frequency of ship–bridge collision is calculated as follows:

(1) $$A_{F}\left(t\right)=\sum_{i=1}^{n}n_{j}P_{Ai}P_{Gi}$$

where $A_{F}\left(t\right)$ is the annual frequency of ship–bridge collision, t is the time range of the evaluation, default as one year, $n_{i}$ is the annual number of the *i* type of ships passing through the bridge, $P_{Ai}$ is the probability of aberrancy of the *i* type of ships, and $P_{Gi}$ is the geometric probability of the aberrant ship of the *i* type of ships (i.e., the probability a ship will hit a bridge pier or superstructure component if it is aberrant in vicinity of bridge). Similar with the Macduff model, both have one geometric probability and one causation probability (in AASHTO, it is called probability of aberrancy).

However, the models are primarily developed and applied for inland waterways with ship traffic in narrow waterways [35]. The values of aberrancy probability of the AASHTO model are mainly based on the US inland river statistics, which may not fit the situations elsewhere. In addition, the model did not consider situations where the ship takes timely collision avoidance measures or get rescued from outside or by anti-collision safety measures. Moreover, the model does not include the collision scenario where the ship loses power and drifts to collide with a bridge, which should be a consideration especially in the sea area nearby cross-sea bridge.

Whilst these models and methods could be used for the ship–bridge collision risk assessment, the lack of concrete consideration of mitigation effects of safety measures limits the application of such models in quantifying collision avoidance probability in scenarios where passive and/or active safety measures are deployed. As more and more safety measures are deployed, the effectiveness and costs associated must be weighed against the benefits gained; the challenge is to find a way to evaluate effectiveness quantitatively and is practical for field use. Therefore, it is necessary to propose new models that consider refined scenarios of deploying safety measures. In addition, the collision probability under each scenario can also be separated to geometric probability and causation probability (in this study, we call it collision avoidance failure probability), which are inspired by the Macduff model and the AASHTO model.

## 3. Evaluation Model

### 3.1. Typical Ship–Bridge Collision Scenarios

The ship–bridge collision scenarios can be divided into two types: powered collision and drifting collision, according to whether the main engine or the steering device of the ship fails. This was based on [36] in which the authors constructed models of collision between ships and offshore wind farms.

The powered collision is caused by the deviations of the ship from the expected track or the expected direction, which may be caused by human factors or technical faults when the ship is still under the control of a crew. The ship failed to adjust the course in time in the process of approaching the non-navigable spans, and finally collided with the bridge. The drifting collision [37] refers to the situation where the crew cannot control the ship and the ship drifts under the influence of the wind, current, and other environmental factors caused by the engine or steering system failure. In the drifting collision model, it is necessary to consider the chance of the ship losing control and drifting into the non-navigable spans, and whether successful rescue operations can be effected during the drifting process.

Then, we constructed the powered collision model and drifting collision model. In each model, the probability was separated into geometric probability and collision avoidance failure probability.

### 3.2. Ship–Bridge Collision Probability Model

The input and output of the ship–bridge collision probability model was determined as a key link in realizing model construction. In this study, the model input mainly refers to the traffic, environment, and bridge factors that affect the ship–bridge collision geometric probability, and active and passive safety measures that affect the ship–bridge collision avoidance probability. On the other hand, the model output refers to the powered collision probability, drifting collision probability, and overall collision probability considering different anti-collision safety measures. According to the analysis of the ship–bridge collision process, the ship–bridge collision scenario includes two types: powered collision and drifting collision. Both should include two modules, such as the geometric collision module and the collision avoidance failure module.

#### 3.2.1. Powered Collision Probability Model

The overall collision probability for the powered collision is the product of geometric probability and collision avoidance failure probability. The geometric probability is whether the ship will sail in the direction of the non-navigable spans of the bridge, which is determined by the location relative to the bridge, position on the fairway of the ship, and the course over ground of the ship. The collision avoidance failure probability is the chance that the crew cannot take the proper collision avoidance measures in time to avoid the collision.

The assumption made for the powered collision is that the sailing speed of the ship is constant over the fairway.

**Figure 2:**
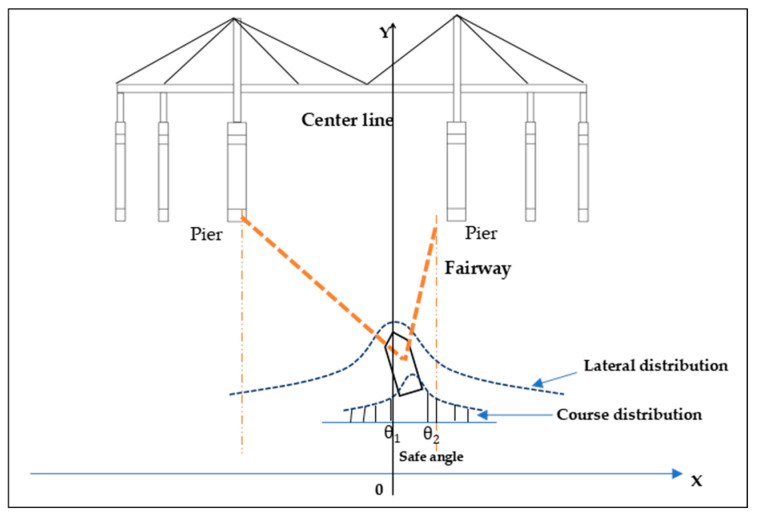
Illustration of the powered collision.

The collision frequency for powered collision for ships on a fairway is calculated using the following equation:

(2) $$F_{cp}=N\cdot \left(1-F_{d}\cdot T\right)\cdot \sum_{y}\sum_{x}\sum_{course}P_{y}\cdot P_{x}\cdot P_{course}\cdot P_{ca1},$$

where $F_{cp}$ is the powered collision frequency, N is the annual traffic volume of ships sailing on the fairway, $F_{d}$ is the frequency of engine failure (per hour), T is the average time ships spend passing through the bridge (hours), $P_{y}$ is the probability of being in position *y* on the fairway, and $P_{x}$ is the probability of having a certain lateral *x* offset from the center of the fairway. Usually, the lateral distribution of ship traffic on the route follows the normal distribution, which is calculated as:

(3) $$f\left(x\right)=\frac{1}{\sqrt{2\pi }\sigma }e^{-\frac{{\left(x-\mu \right)}^{2}}{2\sigma^{2}}},$$

where f(x) is the density probability function of the lateral distribution of the ship on the fairway; the mean value (μ) for the normal distribution is usually assumed to be zero; and the standard deviation (σ) for the normal distribution is estimated from histograms. $P_{course}$ is the probability of following a certain course heading towards the non-navigable spans (Course deviations are assumed to follow a normal distribution too). According to the position of the ship in the route and the scale and position of the bridge spans, the course range of the ship when crossing the bridge safely can be determined (Figure 2). $P_{ca1}$ is the collision avoidance failure probability when the ship is under power.

In [36], the authors compared several models that model the engine failure rate and concluded that the engine failure rate is 2.5 × 10^−4^ per hour. So, $F_{d}$ here is assumed to be 2.5 × 10^−4^ per hour.

#### 3.2.2. Drifting Collision Probability Model

Similar to the powered collision probability submodel, the drifting collision probability can also be divided into geometric probability and collision avoidance failure probability. The geometric probability is whether the ship will drift towards the non-navigable spans of the bridge, and the collision avoidance failure probability is the chance rescue operations cannot be effected during the drifting process.

The assumptions made for the drifting collision are that the drifting direction and the wind direction are in the same direction and the wind direction and velocity are kept constant during the drifting.

The collision frequency for drifting collisions for ships on a fairway is calculated using the following equation:

(4) $$F_{cd}=N\cdot F_{d}\cdot T\cdot \sum_{y}\sum_{x}\sum_{w}P_{y}\cdot P_{x}\cdot (1-P_{w})\cdot P_{ca2},$$

where $F_{cd}$ is the drifting collision frequency; N is the annual traffic volume of ships sailing on the fairway; $F_{d}$ is the frequency of engine failure (per hour); T is the average time the ship spends passing through the bridge (hours); $P_{y}$ is the probability of being in position *y* on the fairway; $P_{x}$ is the probability of having a certain lateral *x* offset from the center of the fairway; $P_{w}$ is the probability that the ship will drift in the direction of the navigable span when the ship is drifting; and $P_{ca2}$ is the collision avoidance failure probability when the ship is without power.

**Figure 3:**
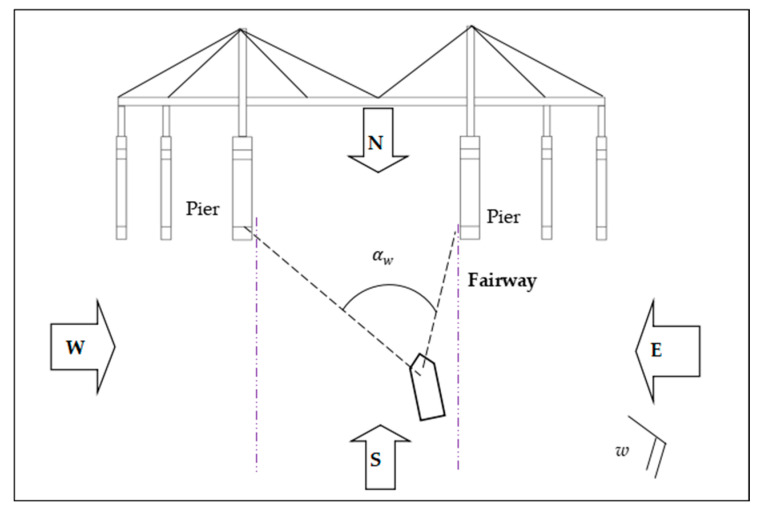
Illustration of the probability of drifting towards the non-navigable spans.

The probability of drifting towards the navigable spans under consideration varies with the ship’s position on the fairway, navigable spans’ position and wind directions. For each ship on the lane the navigable spans are covered by an angle which overlaps with the wind directions as shown in the example in Figure 3 (division in four wind directions).

The probability is therefore dependent on the position of the ship and the frequency of the wind blowing from the different directions. The factor $P_{w}$ is calculated as follows:

(5) $$P_{w}=\sum_{w=1}^{N_{wd}}(\frac{R_{w}\cdot \alpha_{w}}{360/N_{wd}}),$$

where $N_{wd}$ is the number of divisions in different wind directions; $\alpha_{w}$ is the angle which is covered by the navigable spans in the wind direction w; and $R_{w}$ is the frequency for wind from direction w.

### 3.3. Geometric Collision Probability Calculation Based on Monte Carlo Simulation Method

The geometric collision probability model established in this section requires the ship traffic data in the corresponding fairway and the environmental data of the waterways surrounding the bridge as the model input, supplemented by Monte Carlo simulation method to simulate the ship–bridge collision probability.

Automatic Identification System (AIS) data are an essential data source for the estimation of traffic behaviors and navigable capacity of busy waterways. Since 2004, all passenger ships and ships over 300 gross tonnage (GT) have been fitted with AIS transponders [38]. The reporting interval between two consecutive AIS position reports received from the same vessel equipped with AIS Class A receiver are from 2 to 180 s [39]. Since the AIS data record various static and dynamic information about ships, it is widely used for marine traffic safety and ship power analysis. The data analysis in AIS can be conducted to dig out some valuable information from the spatial and temporal ship trajectory data that are constantly accumulated. The dynamic information includes navigation time, longitude, latitude, speed over ground, course over ground and the static information includes Maritime Mobile Service Identity (MMSI, identity of each vessel), vessel type and vessel size, etc. In this contribution, AIS data have been collected to analyze the lateral position distribution and course distribution of the ship in the navigation fairway.

**Figure 4:**
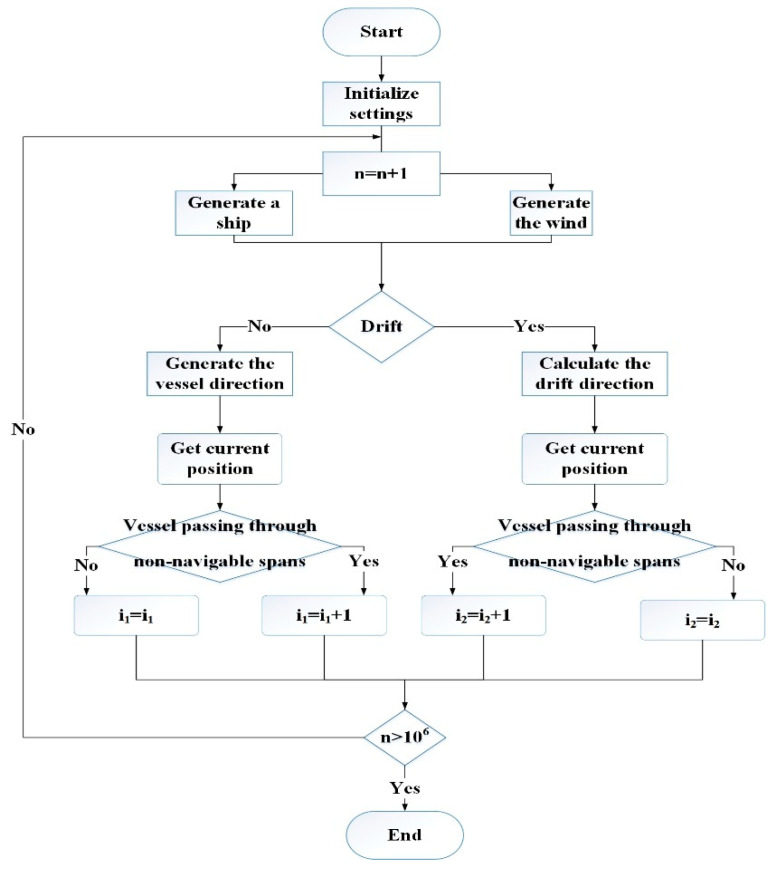
Monte Carlo simulation flow chart to estimate geometric collision probability.

The Monte Carlo simulation is a stochastic simulation originating from probabilistic and statistical theory that can obtain a close numerical solution to the requested problem by setting repeated multiple extraction experiments. This method can be applied to the quantitative analysis of maritime security and has been widely used. The solution flow chart of the ship–bridge collision probability based on the Monte Carlo simulation is shown in Figure 4. The solution process can be divided into five steps described below:

i. Initial condition setting. The initial condition is initialized for these parameters: the fairway location, the bridge location, the position, speed and course distributions of ships, and the wind conditions.
ii. Ship generation. The ship is randomly generated according to the ship’s lateral distribution, navigation speed, and course distribution function.
iii. Environmental conditions’ generation. Wind speed and wind direction are randomly generated according to historical statistics.
iv. Simulation. Simulate the ship’s movement according to generated parameters, record the simulation results.
v. Ship–bridge geometric collision probability calculation. Based on the above collision simulations, it is calculated that the geometric collision frequency of powered collision is $i_{1}$, the geometric collision frequency of drifting collision is $i_{2},$ and the number of simulations is n. The geometric collision probability for powered collision can be calculated as $i_{1}/n$ and the geometric collision probability for drifting collision can be calculated as $i_{2}/n$.

### 3.4. Collision Avoidance Failure Probability Calculation Based on the Bayesian Network Method

Geometric collision probability reflects the basic collision risk of bridges, traffic, and environments in a particular configuration, which can be modeled using probability theory. However, any internal and external intervention, such as crew corrective operations and external safety measures, may alter the collision path and prevent the collision from occurring. This involves a complicated collision avoidance process and complex interactions between crew, traffic, environment as well as passive and active safety measures, and should consider various uncertainty factors and events. Bayesian networks can be used to build models from expert opinion for calculating probability under uncertainty. Thus, we used the Bayesian networks method to calculate the collision avoidance failure probability for the powered collision and drifting collision between ships and bridges.

Bayesian networks (BNs) are a probabilistic graphical model that represents a set of variables and their conditional dependencies via directed acyclic graphs (DAGs). The construction of BNs is mainly divided into three parts: the selection of the nodes, determination of the topology structure, and the determination of the conditional probability table between the nodes. Point selection is based on the analysis of various influencing factors and value domains of this problem. The topology is determined by the analysis of the logical relations between the obtained nodes. Based on this, the conditional probability between the child and parent nodes can be determined based on the historical data, the actual research data, and the expert knowledge [40].

The BNs need to determine both prior and posterior probabilities. The prior probabilities can be obtained through a summary of historical data and expert surveys. The posterior probability can be obtained based on the prior probability, and the computational formula is constructed based on the Bayesian formula. It mainly includes three parts such as prior probability, full probability, and posterior probability. The corresponding posterior probabilities can be obtained by both prior and conditional probabilities. The formula is shown in (6):

(6) $$P\left(B|A\right)=\frac{P(A,B)}{P(A)}=\frac{P(A,B)}{\sum_{B}P(A,B)}=\frac{P\left(A|B\right)P(B)}{\sum_{B}P\left(A|B\right)P(B)},$$

where P(B|A) is the posterior probability, which describes the probability of event B occurring when event A occurs, and P(B) is the prior probability of B, which is calculated by Equation (7).

(7) $$P\left(B\right)=\prod_{i=1}^{n}P(X_{i}|Pa(X_{i}))$$

where $B=\left\{X_{1},X_{2},\ldots X_{n}\right\}$ denotes the set of random variables node $X_{1},X_{2},\ldots X_{n}$; $Pa\left(X_{i}\right)$ denotes the set of $X_{i}$’s parent node; and $P\left(X_{i}|Pa\left(X_{i}\right)\right)$ denotes the conditional probability distribution of the node.

There are several safety measures we consider to evaluate, which include early warning system, tugboat rescue for help (stands for active measures in drifting collision), and arresting cables (stands for passive measures in both powered collision and drifting collision).

#### 3.4.1. Collision Avoidance Failure Probability for Powered Collision

**Figure 5:**
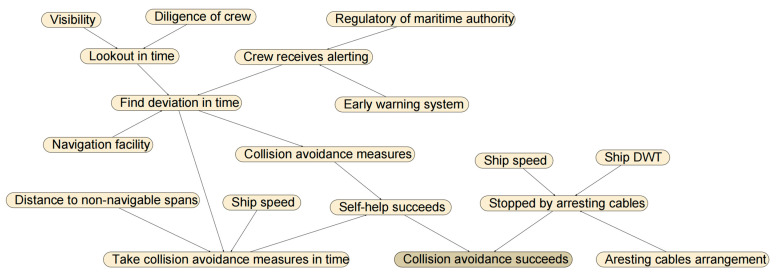
Logical relations of powered collision avoidance.

The factors and logical relations of powered collision avoidance considered in this study are listed in Table 1 and presented in Figure 5, respectively. This is based on the results of a panel discussion of five maritime experts (detailed in case study) and also with reference to [14,23,37].

#### 3.4.2. Collision Avoidance Failure Probability for Drifting Collision

**Figure 6:**
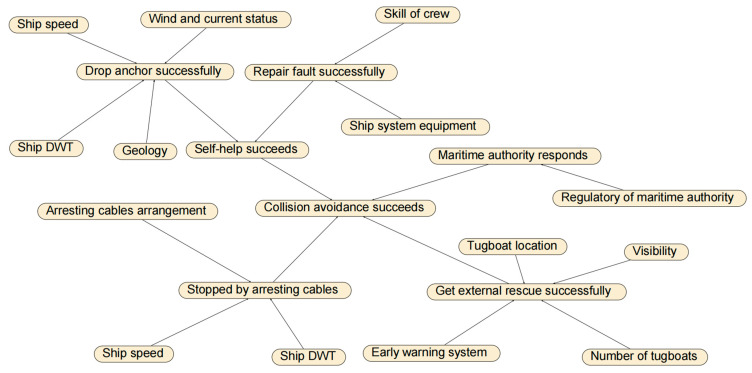
Logical relations of drifting collision avoidance.

The factors and logical relations of drifting collision avoidance considered in this study are listed in Table 2 and presented in Figure 6, respectively. This is based on the results of a panel discussion of five maritime experts (detailed in case study) and also with reference to [14,23,37].

## 4. Evaluation of the Effectiveness of Active and Passive Safety Measures in Jintang Bridge

### 4.1. Study Area and Relative Data

**Figure 7:**
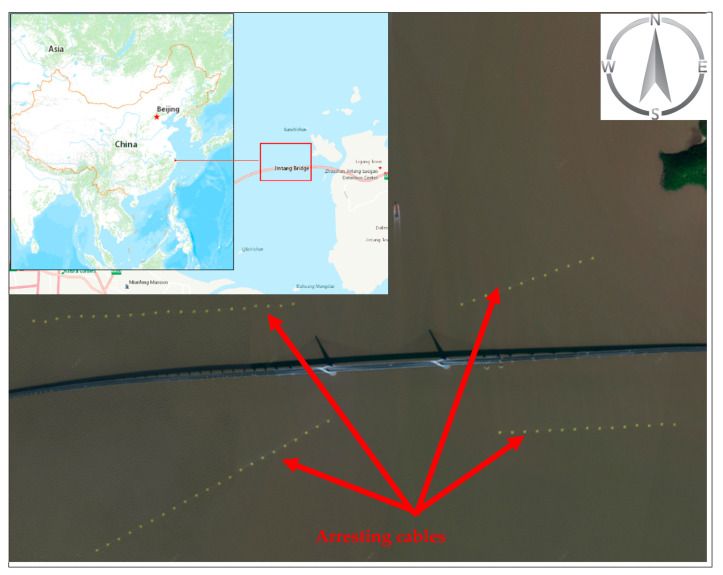
Geographic location and aerial photography of the Jintang Bridge showing the arresting cables’ position (yellow buoys).

**Figure 8:**
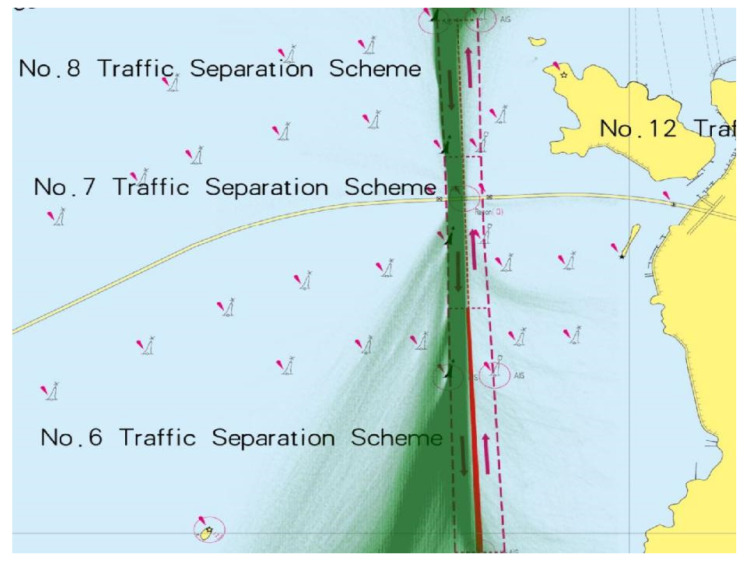
Traffic separation scheme (TSS) in the main navigable span of the Jintang Bridge. The AIS data are also displayed by green points.

Jintang Bridge is located in Zhoushan, Zhejiang Province in China. The bridge consists of the main navigable span, east navigable span, and west navigable span. The majority of traffic volume passed through the main navigable span. The bridge area has a special geographical location, and the water environment is relatively complex. The navigable height of the main navigable span and the navigable clearance width are 51 and 544 m, respectively. Ships of 50,000 dead weight tonnage (DWT) and below are allowed to pass in two directions. This study set the main navigable span of Jintang Bridge as the study area since the traffic flow in the east and west navigable spans is sparse. Arresting cables has been equipped near the navigable span to regulate the navigation behavior of ships and reduce the risk of accidents (Figure 7). In addition, the Jintang Bridge is a highly regulated area implementing strict traffic separation scheme (TSS) to minimize ship collision (Figure 8). The total length and width of the TSS fairway were 5.78 and 0.3 miles, respectively.

We took the place that is 1 nautical mile within the navigable span of the bridge as the study area and calculated both the powered collision probability and drifting collision probability for ships passing through this area under different safety measures.

For convenience, we only studied the collision probability of ships sailing southwards. We collected AIS data in 2020 near the main navigable span to calculate the traffic distribution for the simulation.

The Jintang Bridge is located in a prevalent windy area with subtropical monsoon climate being the wind quite predictable, as shown in Table 3. Adverse factors threaten the navigation safety of the surrounding ships and the safety of the bridge itself. It is necessary to evaluate the effectiveness of different safety measures in preventing ship–bridge collision for Jintang Bridge.

### 4.2. Geometric Collision Probability Analysis

In the example case of the Jintang Bridge, we considered four different configurations in the function of the measures adopted: active measures (setting risk detecting and alarming systems and rescue tugboats for help), passive measures (setting arresting cables), neither active nor passive measures, and both active and passive measures. The number of total traffic trajectories southwards is 28,758, which covers one year.

Analogous to the traffic distribution, the lateral distribution (m) follows a normal distribution N(0,71). Moreover, the ship speed (kn) and ship course follow a normal distribution N(7.72, 2.43) and N(177.3, 3.88), respectively.

Considering these factors and based on the Monte Carlo simulation method mentioned in Section 2.2, the geometric collision probability was calculated, which was 2.469 × 10^−2^ and 2.1106 × 10^−5^ for powered collision and drifting collision, respectively.

### 4.3. Collision Avoidance Failure Probability Analysis

A focus group meeting was arranged in January 2022, where five maritime experts from the Maritime Safety Administration (Senior Official), Bridge Engineering Company (Senior Engineer), Search and Rescue Company (Captain), Shipping Company (Captain), and university (Professor) attended. Expertise fields include navigation, maritime safety, maritime search and rescue, and risk evaluation. The moderator gives an introduction to the case study, safety measures, and an overview of the potential factors considered, and led the focus group to discuss prospective factors to be included in the modeling framework.

This was followed by a Delphi study where the experts’ scores on the conditional probability of each node for the BN model with explanatory comments were collected, and feed-backed to the panel, so the experts can reconsider and modify their answers based on others’ opinions. Through the process, a certain degree of consensus was achieved. The panel members were then asked to agree on a final score on the conditional probability of each node based on their individual answers together with the statistical results from the previous round.

**Figure 9:**
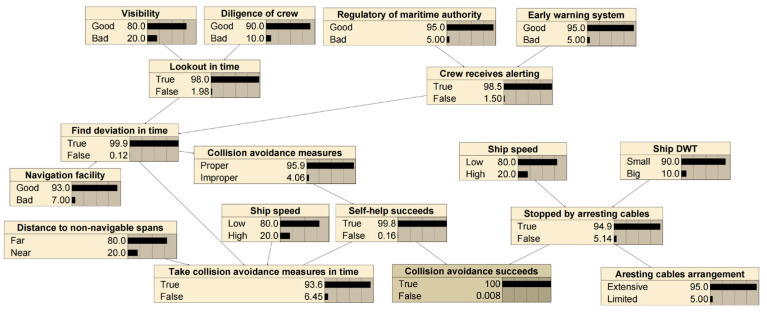
BNs model of the powered collision avoidance.

**Figure 10:**
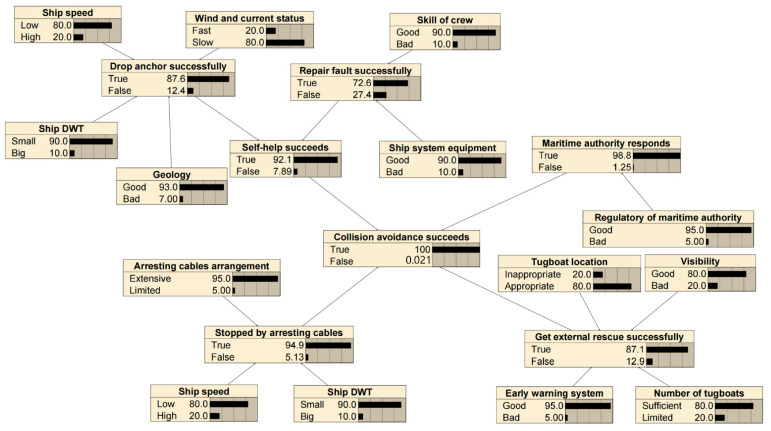
BNs model of the drifting collision avoidance.

Finally, we set the corresponding BNs (see Figure 9 and Figure 10) and calculated the collision avoidance failure probability using the Netica BN software by Norsys Software Corporation (http://www.norsys.com/, accessed on 10 January 2022). Then, we set active measures and passive measures probability to 100% of “False”, respectively. Subsequently, the probability under different safety measures can be obtained. The results of the BNs are shown in Table 4 and Table 5 for the powered and drifting collision avoidance, respectively.

### 4.4. Effectiveness of Active and Passive Safety Measures

**Figure 11:**
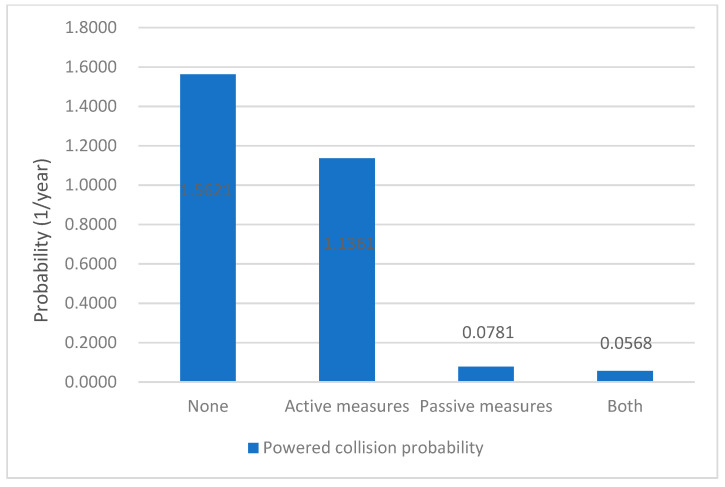
Powered collision probability under different safety measures.

**Figure 12:**
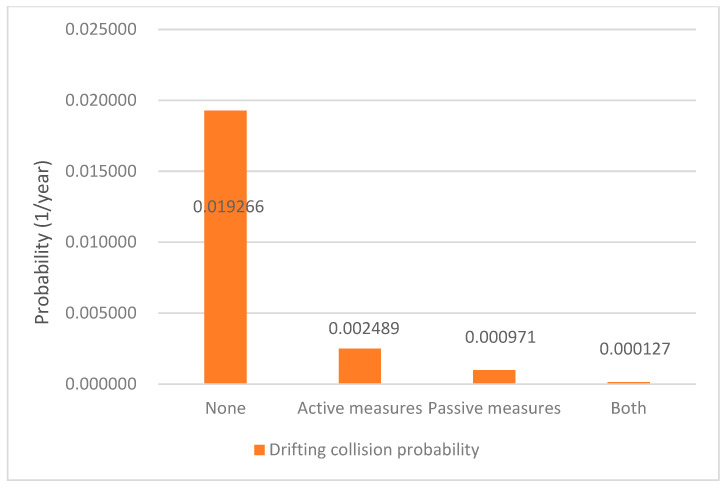
Drifting collision probability under different safety measures.

The results of the annual collision probability of our case for both powered collision and drifting collision are shown in Figure 11 and Figure 12, respectively, in function of the measures considered.

For powered collision, if no measures were taken, the ship–bridge collision probability was 1.56. Moreover, if active, passive, or both measures were taken, the ship–bridge collision probabilities were 1.14, dropped to 7.81 × 10^−2^, and 5.68 × 10^−2^, respectively. Figure 11 shows that in powered collision, the passive measures are more effective in powered collision than that in active measures.

For drifting collision, if no measures were taken, the ship–bridge collision probability was 1.93 × 10^−2^. If active, passive, or both measures were applied, the ship–bridge collision probability was 2.49 × 10^−3^, dropped to 9.71 × 10^−4^, and 1.27 × 10^−4^, respectively. In this sense, Figure 12 shows that both active and passive safety measures are effective in preventing the drifting collision. However, the passive measures are more effective compared to active measures.

Consequently, for the case of Jintang, four key points can be drawn. First, the passive measures are more effective than the active measures in preventing the powered collision and drifting collision. Second, for preventing powered collision, the active measures have no significant effect. Third, for preventing drifting collision, the effect of the passive measures and active measures are roughly of the same order of magnitude. Lastly, every collision probability can be reduced to the lowest level if taking both active and passive measures.

## 5. Discussion and Conclusions

This paper proposed a novel method for evaluating the effectiveness of safety measures to avoid ship–bridge collisions. Both the natural ship–bridge collision risk (geometric collision probability) and effectiveness of remedy safety measures (collision avoidance probability) were considered in an integrated framework. It is based on establishing a ship–bridge collision probability model consisting of two submodels: powered collision probability submodel and drifting collision probability submodel. Both models consider the geometric collision probability and collision avoidance failure probability. We used the Monte Carlo simulation method and Bayesian networks to solve the geometric collision probability and the ship collision avoidance failure probability, respectively. Based on the massive AIS data, we applied the collision probability model at Jintang Bridge as an example of a highly crowded route. Compared to other models (e.g., [25,28,29]), our model considers refined collision scenarios and evaluates the collision avoidance considering active and passive measures. This means that our study aims to consider the effects of the ship–bridge collision probability with different kinds of measures, which are not included in previous contributions, for the first time. Therefore, models that considered AIS data, the ship maneuvering simulator, and the Monte Carlo method to calculate the accident probabilities in marine traffic near bridges may benefit from the inclusion of active and passive measures [31,32]. From our study case, we concluded that both the active and passive safety measures are effective in preventing the ship–bridge collision and the passive measures provide a higher contribution. This confirms the necessity of setting arresting cables around Jintang Bridge. Passive measures are preferred because of the disadvantages of active measures in preventing the ship–bridge collision. For example, dispatching a rescue tugboat was ineffective because the active collision risk detecting system cannot respond as fast as the situation needs especially during powered collision (i.e., the ship–bridge collision can happen at any moment).

From a practical perspective based on the Jintang Bridge case, the active measures should be upgraded to perform a faster response. If an active response is deployed properly, active safety measures can be provided to the bridge with equivalent protection as passive measures against collision risks. Even though the result was focused on the Jintang Bridge (with its characteristics), the methodology and results may be useful to be applied in similar other cross-sea bridges.

Our study has shown a promising evaluation of the effectiveness of active and passive safety measures in preventing ship–bridge collisions, indicating that this research has a substantial contribution for the evaluation of bridge protection measures. However, there are many challenges that remain unexplored. For example, the cost of setting passive measures must be considered because, for instance, the implementation of arresting cables is costly. From a methodological perspective, using the Monte Carlo simulation and Bayesian networks allows the calculation of the collision probability, but there is still a gap in the integration of the environmental variables (such as water waves in an eventual operational system). For further studies, we plan to include the use of navigation simulators to simulate more realistic scenarios to calculate accurate ship–bridge collision probability under different safety measures.

## Figures and Tables

**Table 1:** Factors affecting the powered collision avoidance.

Factor	State	
Diligence of crew	Good	Bad
Visibility	Good	Bad
Lookout in time	True	False
Navigation facility	Good	Bad
Distance to non-navigable spans	Far	Near
Ship speed	Slow	Fast
Find deviation in time	True	False
Take collision avoidance measures in time	True	False
Collision avoidance measures	Proper	Improper
Ship DWT	Big	Small
Regulatory of maritime authority	Good	Bad
Early warning system	Good	Bad
Arresting cables arrangement	Extensive	Limited
Self-help succeeds	True	False
Stopped by arresting cables (passive measure)	True	False
Crew receives alerting (active measure)	True	False
Collision avoidance succeeds	True	False

**Table 2:** Factors affecting the drifting collision avoidance.

Factor	State	
Geology	Good	Bad
Wind and current status	Fast	Slow
Ship DWT	Small	Big
Ship speed	Low	High
Skill of crew	Good	Bad
Ship system equipment	Good	Bad
Visibility	Good	Bad
Arresting cables arrangement	Extensive	Limited
Drop anchor successfully	True	False
Repair fault successfully	True	False
Self-help succeeds	True	False
Regulatory of maritime authority	Good	Bad
Early warning system	Good	Bad
Number of tugboats	Sufficient	Limited
Tugboat location	Appropriate	Inappropriate
Maritime authority responds	True	False
Get external rescue successfully (active measure)	True	False
Stopped by arresting cables (passive measure)	True	False
Collision avoidance succeeds	True	False

**Table 3:** Wind direction frequency and averaged wind speed.

Wind Direction	Average Speed (m/s)	Frequency (%)
N	9.1	10
NNE	7.1	8
NE	6.5	6
ENE	5.7	5
E	4.8	5
ESE	6	6
SE	6.4	6
SSE	7.8	13
S	7.6	8
SSW	6.2	3
SW	3.5	2
WSW	3.8	2
W	5.1	3
WNW	7.6	4
NW	8.3	4
NNW	9	9
C		6

**Table 4:** Collision avoidance failure probability for powered collision.

Kind of Measures	Collision Avoidance Failure Probability
No measures	2.2 × 10^−3^
Active measures	1.6 × 10^−3^
Passive measures	1.1 × 10^−4^
Both measures	8 × 10^−5^

**Table 5:** Collision avoidance failure probability for drifting collision.

Kind of Measures	Collision Avoidance Failure Probability
No measures	3.21 × 10^−2^
Active measures	4.1 × 10^−3^
Passive measures	1.6 × 10^−3^
Both measures	2.1 × 10^−4^

## Data Availability

For contractual and privacy reasons, the raw AIS data is not available. Upon request, the authors can provide a sample of data for replication.

## References

[1] American Association of State Highway and Transportation Officials (2012). AASHTO LRFD Bridge Design Specifications.

[2] Gong T. (2010). Research on Possibilities of Ship-Bridge Collision Accident. Master’s Thesis.

[3] Accident Investigation Report of “UNIVERSAL MK 2017” Contacting Anti-Collision Pile of Hong Kong-Zhuhai-Macao Bridge. https://www.msa.gov.cn/public/documents/document/mdqy/otay/~edisp/20191223042902163.pdf.

[4] Chen Y. (2016). Study on the Visualization of Collision Risk of Sutong Yangtze River Highway Bridge Based on AIS Data. Master’s Thesis.

[5] Zhang W., Jin X., Wang J. (2014). Numerical analysis of ship-bridge collision’s influences on the running safety of moving rail train. Ships Offshore Struct..

[6] Liu Y., Xiao Y. (2014). Risk Degree of Ship-bridge Collision based on Theory of Ship Collision Avoidance. Int. J. Control Autom..

[7] Shi Y. (2020). Study on the Active Early Warning System of Vessel Control in Bridge Area. Henan Sci. Technol..

[8] Moan T., Amdahl J., Ersdal G. (2019). Assessment of ship impact risk to offshore structures—New NORSOK N-003 guidelines. Mar. Struct..

[9] Pietrzykowski Z. (2008). Ship’s fuzzy domain—A criterion for navigational safety in narrow fairways. J. Navig..

[10] Goodwin E.M. (1975). A Statistical Study of Ship Domains. J. Navig..

[11] Fujii Y., Tanaka K. (1971). Traffic Capacity. J. Navig..

[12] Fujii Y., Yamanouchi H., Mizuki N. (1974). Some factors affecting the frequency of accidents in marine traffic. II: The probability of stranding. III: The effect of darkness on the probability of stranding. J. Navig..

[13] Macduff T. (1974). Probability of vessel collisions. Ocean Ind..

[14] Hänninen M., Kujala P. (2010). The effects of causation probability on the ship collision statistics in the Gulf of Finland. TransNav Int. J. Mar. Navig. Saf. Sea Transp..

[15] Ylitalo J. (2009). Ship-Ship Collision Probability of the Crossing Area between Helsinki and Tallinn.

[16] Cem Kuzu A., Akyuz E., Arslan O. (2019). Application of Fuzzy Fault Tree Analysis (FFTA) to maritime industry: A risk analysing of ship mooring operation. Ocean Eng..

[17] Pedersen P.T. (1995). Collision and grounding mechanics. Proc. WEMT.

[18] Fowler T.G., Sørgård E. (2000). Modeling Ship Transportation Risk. Risk Anal..

[19] Thanh N., Park Y.-S., Park J.-S., Jeong J.-Y. (2013). Developing a Program to Pre-process AIS Data and applying to Vung Tau Waterway in Vietnam—Based on the IWRAP Mk2 program. J. Korean Soc. Mar. Environ. Saf..

[20] Wu B., Yip T.L., Yan X., Guedes Soares C. (2019). Fuzzy logic based approach for ship-bridge collision alert system. Ocean Eng..

[21] Pedersen P.T., Chen J., Zhu L. (2020). Design of bridges against ship collisions. Mar. Struct..

[22] Li T. (2019). Research on Real-Time Collision Risk Index for Inland Vessel-Buoy Based on AIS. Master’s Thesis.

[23] Ou Y. (2014). Study on Ship Collision Risk Assessment of Jiashao Bridge. Master’s Thesis.

[24] Xia H. (2021). Navigational risk analysis based on GIS spatiotemporal trajectory mining: A case study in Nanjing Yangtze River Bridge waters. Arab. J. Geosci..

[25] Lu K., Chen X., Shen H. (2021). Summary of Research on Ship-bridge Collision. Port Eng. Technol..

[26] Larsen O.D. (1993). Ship Collision with Bridges: The Interaction between Vessel Traffic and Bridge Structures.

[27] Gluver H., Olsen D. (1998). Ship Collision Analysis.

[28] Koh H.M., Lim J.H., Kim H., Yi J., Park W., Song J. (2017). Reliability-based structural design framework against accidental loads—Ship collision. Struct. Infrastruct. Eng..

[29] Zhang W., Liu S., Luo W., Wang L., Geng B., Zheng Z. (2020). A New Approach for Probabilistic Risk Assessment of Ship Collision with Riverside Bridges. Adv. Civ. Eng..

[30] Yu G., Gan W. (2021). Study on the Probability Model of Ship-Bridge Collision. IOP Conf. Ser. Earth Environ. Sci..

[31] Hörteborn A., Ringsberg J.W. (2021). A method for risk analysis of ship collisions with stationary infrastructure using AIS data and a ship manoeuvring simulator. Ocean Eng..

[32] Amdahl J., Ehlers S., Leira B.J. (2013). Collision and Grounding of Ships and Offshore Structures.

[33] Park W., Lim J.H., Koh H.M. (2013). Estimation of probabilistic scenario-based design load for extreme events. KSCE J. Civ. Eng..

[34] Yu Q., Liu K., Yang Z., Wang H., Yang Z. (2021). Geometrical risk evaluation of the collisions between ships and offshore installations using rule-based Bayesian reasoning. Reliab. Eng. Syst. Saf..

[35] Hörteborn A. (2021). Ship Behaviour and Ship-Bridge Allision Analysis. Licentiate Thesis.

[36] Ellis J., Forsman B., Huffmeier J., Johansson J. (2008). Methodology for Assessing Risks to Ship Traffic from Offshore Wind Farm.

[37] Nie Y. (2019). Research on Ship Collision Risk for an Offshore Wind Farm. Master’s Thesis.

[38] Liu C., Liu J., Zhou X., Zhao Z., Liu Z. (2020). AIS data-driven approach to estimate navigable capacity of busy waterways focusing on ships entering and leaving port. Ocean Eng..

[39] Jaskólski K., Marchel Ł., Felski A., Jaskólski M., Specht M. (2021). Automatic Identification System (AIS) Dynamic Data Integrity Monitoring and Trajectory Tracking Based on the Simultaneous Localization and Mapping (SLAM) Process Model. Sensors.

[40] Chai T., Weng J., De-Qi X. (2017). Development of a quantitative risk assessment model for ship collisions in fairways. Saf. Sci..

